# Guidelines for the treatment of dysentery (shigellosis): a systematic review of the evidence

**DOI:** 10.1080/20469047.2017.1409454

**Published:** 2018-05-23

**Authors:** Phoebe C. M. Williams, James A. Berkley

**Affiliations:** a Nuffield Department of Clinical Medicine, The University of Oxford, Oxford, UK; b Kenya Medical Research Institute (KEMRI)/Wellcome Trust Research Programme, Kilifi, Kenya; c The Childhood Acute Illness and Nutrition Network (CHAIN), Kilifi, Kenya; d Centre for Tropical Medicine and Global Health, Nuffield Department of Medicine, The University of Oxford, Oxford, UK

**Keywords:** Dysentery, shigellosis, shigella, antibiotics, antimicrobial resistance, treatment guidelines

## Abstract

**Background**: Shigella remains the primary cause of diarrhoea in paediatric patients worldwide and accounts for up to 40,000 deaths per year. Current guidelines for the treatment of shigellosis are based on data which are over a decade old. In an era of increasing antimicrobial resistance, an updated review of the appropriate empirical therapy for shigellosis in children is necessary, taking into account susceptibility patterns, cost and the risk of adverse events.

**Methods**: A systematic review of the current published literature on the treatment of shigella dysentery was undertaken in accordance with the Preferred Reporting Items for Systematic Reviews and Meta-Analyses (PRISMA).

**Results**: The initial search produced 131 results, of which nine studies met the inclusion criteria. The quality of the studies was assessed as per the Grading of Recommendations Assessment, Development and Evaluation (GRADE) guidelines. International guidelines were also reviewed. There is a lack of current research regarding the clinical treatment of shigellosis in paediatric and adult patients, despite rising antimicrobial resistance worldwide. In particular, there is a lack of studies assessing the non-susceptibility of community-acquired strains, with almost all published research pertaining to microbiological data from hospital-based settings.

**Discussion**: Current WHO guidelines support the use of fluoroquinolones (first-line), β-lactams (second-line) and cephalosporins (second-line) which accords with currently available evidence and other international guidelines, and there is no strong evidence for changing this guidance. Azithromycin is appropriate as a second-line therapy in regions where the rate of non-susceptibility of ciprofloxacin is known to be high, and research suggests that, from a cardiac point of view, azithromycin is safer than other macrolide antibiotics. Cefixime is also a reasonable alternative, although its use must be weighed against the risk of dissemination of extended-spectrum β-lactamase-producing organisms.

## Introduction

Shigella is a Gram-negative, non-motile bacillus belonging to the enterobacteriacae family of which four species exist: *S. dysenteriae, S. flexneri, S. boydii* and *S. sonnei* (designated as serogroups A, B, C and D, respectively) with multiple serotypes. Of the estimated 165 million shigella diarrhoeal episodes every year, 99% of cases occur in low- and middle-income countries (LMIC), mainly (69%) in children [1]. Shigella has recently been identified as the leading pathogen causing childhood diarrhoea worldwide, and has been estimated to be responsible for 1.1 million deaths per year, 61% of which are children <5 years of age [2,3].

Because of overcrowding and poor sanitation, shigellosis occurs predominantly in LMIC. Infants, non-breast fed or malnourished children and adults >50 years have a more severe illness and a greater risk of death [4]. Acquired immunity to shigella is serotype-specific. While *S. boydii* and *S. sonnei* usually cause a relatively mild illness (watery or bloody diarrhoea only), *S. flexneri* and *S. dysenteriae* are chiefly responsible for endemic and epidemic shigellosis, respectively, in developing countries, with high transmission rates and significant case fatality. *S. dysenteriae* (Type 1, also known as Shiga bacillus) is capable of causing a more severe and prolonged illness owing to the production of a potent cytotoxin (Shiga) which is associated with the development of haemolytic-uraemic syndrome [5]. Other complications of shigellosis include sepsis, rectal prolapse, arthralgia, intestinal perforation, toxic megacolon, electrolyte imbalance, seizures and leukaemoid reactions [1,2].

The species distribution of shigella infection varies globally. While *S. sonnei* is the predominant species worldwide, *S. flexneri* is more prominent in low-income settings in Africa and Asia [1,4] while the less virulent *S. sonnei* predominates in higher-income settings [5,6]. Transmission occurs via a number of mechanisms – the faecal/oral route, person-to-person contact, household flies, infected water, or inanimate objects following exposure to as few as 10–100 organisms [3,6]. Once infected, all shigella species multiply and cause acute bloody diarrhoea by invading the colonic epithelium where pro-inflammatory cytokines are released, and the subsequent inflammatory reaction (recruiting a number of polymorphonuclear cells) destroys the epithelial cells which line the gut mucosa, allowing for further direct invasion by shigella.

The resultant infectious diarrhoea causes a loss of water and electrolytes with a clinical picture of abdominal cramping, fever, and bloody/mucoid stools. Stool microscopy – a cheap, rapid and simple diagnostic test – demonstrates numerous polymorphonuclear cells on methylene blue stain; however, microbiological culture is required to differentiate between shigella and other causes of colitis [7]. Multiplex polymerase chain reaction (PCR) platforms for the detection of shigella are commercially available but in most health-care settings their availability is limited.

With effective antibiotic therapy, clinical improvement occurs within 48 h, resulting in a decreased risk of serious complications and death, shorter duration of symptoms, and the elimination of shigella from the stool. This results in diminished transmission of infection by decreasing the duration of faecal carriage from approximately 4 weeks to 3 days, conferring significant public health benefits [8,9]. Current guidelines for treating shigella were published by WHO in 2005 and they recommend ciprofloxacin as the first-line treatment (Table 1) [4]. The guidelines also noted that pivmecillinam (amdinocillin pivoxil) and ceftriaxone were ‘the only antimicrobials that are usually effective for the treatment of multi-resistant strains of Shigella in all age groups’, yet their usage is limited by their high cost and formulation (four times daily dosing for pivmecillinam, and parenteral administration for ceftriaxone). Pivmecillinam and ceftriaxone were therefore only listed for use when local strains of shigella are known to be resistant to ciprofloxacin. Azithromycin was included as a second-line therapy for adults. The 2005 guidelines also listed antimicrobials which should not be used to treat shigellosis owing to their poor mucosal penetration or increasing antimicrobial resistance (Table 2). The more recently published 2013 WHO Pocketbook of Hospital Care for Children included a chapter on the treatment of shigella dysentery, with recommendations which were the same as in the 2005 guidelines [10].

**Table 1.** 2005 WHO guidelines: antimicrobials for treatment of shigellosis (adapted) [4].

Antimicrobial	Treatment schedule for children	Limitations
1st-line: ciprofloxacin	15 mg/kg orally twice daily for 3 days	Expensive
		Resistance emerging
		Drug interactions
2nd-line: pivmecillinam	20 mg/kg orally 4 times daily for 5 days	Cost
		No paediatric formulation
		Four times daily dosing
		Resistance emerging
OR*: ceftriaxone	50–100 mg/kg intramuscular injection for 2–5 days	Requires parenteral administration
		Generates antimicrobial resistance
OR: (for adults) azithromycin	6–20 mg/kg, orally once daily for 1–5 days	Cost
		Drug interactions
		Resistance emerges rapidly, spreads to other bacteria

* Ceftriaxone is listed as an alternative therapy ‘only for use when local strains of shigella are known to be resistant to ciprofloxacin’.

**Table 2.** Antimicrobials highlighted as inappropriate for shigellosis in the 2005 WHO guidelines [4].

Antimicrobial	Rationale for not prescribing
Ampicillin	Antimicrobial resistance
Chloramphenicol	Antimicrobial resistance
Co-trimoxazole	Antimicrobial resistance
Tetracyclines	Antimicrobial resistance
Nalidixic acid	Antimicrobial resistance; cross-resistance to ciprofloxacin observed (MIC increased)
Nitrofurans (nitrofurantoin, furazolidone)	Penetrate the intestinal mucosa poorly
Oral aminoglycosides (gentamicin, kanamycin)	Penetrate the intestinal mucosa poorly
1st- and 2nd-generation cephalosporins (cefazolin, cephalotin, cefaclor, cefoxitin)	Penetrate the intestinal mucosa poorly
Amoxicillin	Penetrates the intestinal mucosa poorly

Current guidelines are therefore based on evidence which is increasingly outdated. In view of changing patterns of resistance to antimicrobials worldwide, this systematic review was undertaken to evaluate the current international literature on the treatment of shigellosis in children.

## Methods

A systematic search for systematic reviews, meta-analyses, multi-centre studies and randomised controlled trials of antibiotic therapy was undertaken using the MeSH search terms ‘Shigella’, ‘dysentery’, ‘antibiotics’ and ‘antimicrobials’. The databases EMBASE, Cochrane database of systematic review and Pubmed were searched. To ensure accurate and up-to-date information on antimicrobial non-susceptibility patterns, the search was limited to trials in humans published since 2005. Inclusion and exclusion criteria are listed in Table 3.

**Table 3.** Inclusion and exclusion criteria.

Inclusion criteria	Exclusion criteria
• Systematic review, randomised controlled trial or multi-centre study investigating clinical treatment options and outcomes for shigellosis • Paediatric-specific information included • Where resistance patterns were investigated, information on antimicrobial testing methodologies documented	• Published before 2005 • Not pertaining to treatment in humans • Data pertaining to carriage rates only

Initially, the search was restricted to studies in the paediatric population but published research in this age group was limited and the search was, therefore, expanded to include all ages. International clinical practice guidelines were also reviewed, including the Infectious Diseases Society of America (IDSA), BMJ Clinical Evidence, the American Academy of Pediatrics and Therapeutic Guidelines (Australia).

## Results

**Figure 1.**
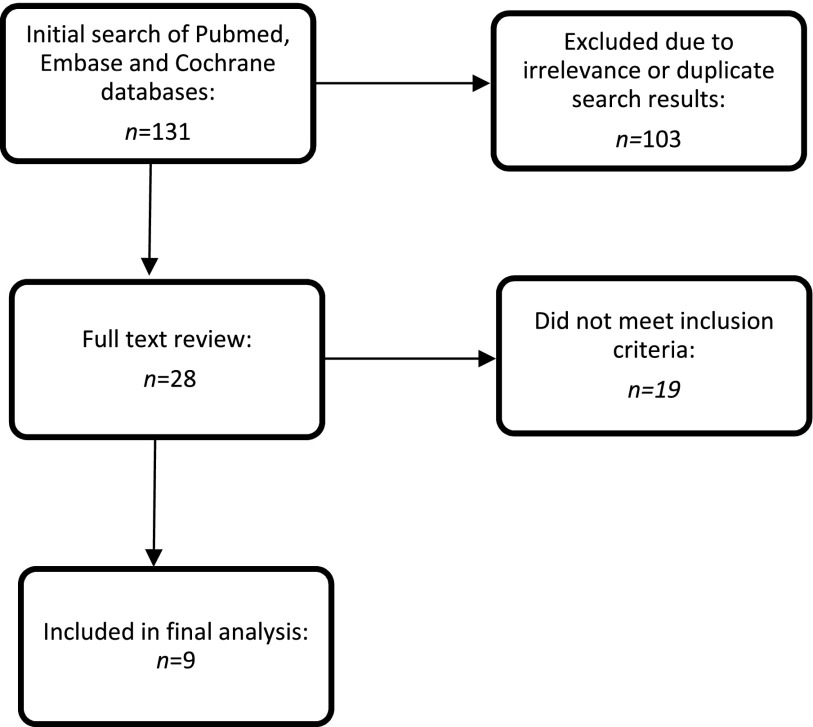
Search strategy.

The initial search produced 131 results (Figure 1), 28 of which qualified for full-text review. Ultimately, nine studies met the inclusion criteria and were abstracted as detailed in Appendix 1. The quality of the studies was assessed as per the Grading of Recommendations Assessment, Development and Evaluation (GRADE) guidelines (see Appendix 1 for description of methodologies) [11].

### Characteristics of included studies

Of the papers eligible for inclusion, six studies were systematic reviews and meta-analyses (conducted across an international setting). One, a multi-centre study, evaluated 600,000 cases of shigella diarrhoea in six Asian countries. Two papers were based on data collated from a multi-centre randomised trial in Vietnam.

Four papers were classified as high-quality evidence, three as moderate-quality evidence and the two papers based on data investigating a multi-centre trial in Vietnam were classified as low-quality evidence.

Two systematic reviews exclusively assessed paediatric antimicrobial response, while the remainder included adults and children in their population. The papers based on a multi-centre trial in Vietnam investigated clinical outcomes in children < 16 years.

### Evidence for current WHO recommendations: ciprofloxacin, pivmecillinam and ceftriaxone

A systematic review in 2013 [12] used CHERG standard rules [13] to analyse 48 high-quality randomised controlled trials in children aged <16 years mainly in low- and middle-income countries (LMIC), seven of which were ultimately eligible for inclusion. It demonstrated that current WHO guidelines for treatment with either ciprofloxacin, pivmecillinam or ceftriaxone reduced clinical failure rates (which the authors postulated were a proxy for shigella deaths) by 82% (95% CI 67–99%). It was reported that ciprofloxacin, pivmecillinam or ceftriaxone successfully cleared shigella pathogens in 96% of cases (95% CI 88–99%). The authors concluded that there is strong evidence that current antimicrobial guidelines are effective in preventing serious mortality and morbidity. Of note, 98% of the trials reviewed were undertaken in LMIC which increases the generalisability and applicability of the findings. However, all studies were hospital-based which limits the information available on resistance patterns for shigella infections typically treated within the community.

Another systematic review in 2010 [14] assessed ciprofloxacin, pivmecillinam or ceftriaxone for children in LMIC (limited to hospital-based settings) using CHERG rules to document clinical failure rates of 0.1% (95% CI-0.2–0.5%). It concluded that the antimicrobial antibiotics currently recommended by WHO are effective from both a clinical and bacteriological point of view.

A third systematic review [8] assessed changing patterns of resistance to ciprofloxacin (alone) and found resistance to be increasing in the Asia–Africa regions (analysed together), from 0.6% in 1998–2000 (95% CI 0.2–1.3%) to 29.1% in 2007–2009 (95% CI 0.9–74.8%) – a 49-fold increase over 12 years. This increase in resistance was significantly above the (minimal) increase documented in the Europe–America region which had reached only 0.6% (95% CI 0.2–1.2%) by 2007–2009. Of note, the review also found higher resistance patterns in children, with respective rates (globally) of 7.5% (95% CI 4.3–11.5%) in paediatric patients *vs* 3.6% in adults (95% CI 2.2–5.3%).

The same authors conducted a review which compared resistance to third-generation cephalosporins (ceftriaxone, cefotaxime and ceftazidime) between 1999 and 2012 [15]: it found markedly increased resistance in the Asia–Africa region [with ceftriaxone resistance reaching 14.2% (95% CI 3.9–29.4%) by 2012]. However, both studies lacked data pertaining to patient outcomes. The authors concluded that ceftriaxone and cefotaxime may not be appropriate for shigellosis in Asia–Africa.

Assessing the Asia region independently, a 2006 multi-centre study (of 2927 shigella isolates in children and adults) in Bangladesh, China, Pakistan, Indonesia, Thailand and Vietnam documented ciprofloxacin-resistant *S. flexneri* isolates in China (18/305, 6%), Pakistan (8/242, 3%) and Vietnam (5/282, 2%) [9].

### Evidence for alternative antibiotic treatment options

In view of the research above documenting increasing resistance to ciprofloxacin and ceftriaxone, the international literature was reviewed for alternative antimicrobials that might be used for shigella dysentery. Previously effective agents (including nalidixic acid, amoxicillin and co-trimoxazole) have been removed from the WHO guidelines on dysentery because of extensive resistance, a decision which continues to be supported in view of the evidence above.

A 2010 Cochrane review [2] investigated antibiotic therapy for shigella dysentery but found no superior efficacy when comparing fluoroquinolones, β-lactams or macrolides. The authors noted that the current practice of presumptively treating shigella dysentery with antibiotics should continue because of the public health benefits conferred, but that no specific antibiotic, or antibiotic class, is universally effective for shigella. However, this study included several randomised controlled trials of low- to moderate-quality (many of which were conducted before 1990) and probably do not reflect current resistance patterns.

#### Aminoglycosides

A 2013 systematic review [16] which assessed worldwide patterns of aminoglycoside resistance in shigella (between 1999 and 2010) documented increasing levels of *in vitro* gentamicin resistance in the Asia–Africa region which reached 32.4% (95% CI 17.87–48.91%) in 2005–2007. Resistance to gentamicin, kanamycin and amikacin was higher in children than in adults. The 2010 Cochrane review outlined above further supported the ineffectiveness of aminoglycosides which tend to have poor absorption when administered orally, further limiting their usefulness.

#### Fluoroquinolones

Gatifloxacin, a fourth-generation fluoroquinolone, was investigated as an alternative therapy in a multi-centre randomised trial assessing the efficacy of gatifloxacin *vs* ciprofloxacin for shigellosis in Vietnamese children between 2006 and 2008 [17]. No superiority to gatifloxacin was found in terms of clinical failure rates which were similar in both groups (gatifloxacin 12% *vs* 11% for ciprofloxacin, *p* = 0.72), with gatifloxacin showing similar efficacy in the treatment of paediatric dysentery [2]. However, while gatifloxacin might be more convenient than ciprofloxacin owing to its longer half-life (allowing administration once daily rather than twice daily as required for ciprofloxacin), retrospective review of clinical outcomes in patients treated with gatifloxacin revealed significantly poorer clinical outcomes than in those treated with ciprofloxacin, regardless of isolate minimum inhibitory concentrations (MIC). Overall, no association between MIC and clinical outcome in paediatric shigellosis was found [17].

#### Macrolides

Azithromycin, a macrolide antibiotic, is listed as an alternative second-line therapy for adults in current WHO guidelines as well as in most international guidelines (for both paediatric and adult patients). To date, no trials comparing the efficacy of azithromycin *vs* ciprofloxacin in children have been published. In the late 1990s, azithromycin was found to be as effective as ciprofloxacin in adults in Kenya and Bangladesh [18]. In Tanzanian adults in 2004–2005, 90% of shigella strains isolated were *S. flexneri* and all were reported to be sensitive *in vitro* to ciprofloxacin, nalidixic acid and cefuroxime, while 98% were sensitive to azithromycin. By 2010/2011 in Dhaka, Bangladesh, *in vitro* susceptibilities to ciprofloxacin, pevmecillinam, azithromycin and ceftriaxone were 65, 50, 74 and 95%, respectively [19]. More recently, however, increasing reports of azithromycin-resistant strains of shigella spp. have been documented, including by the Centers for Disease Control and Prevention (CDC) [20–24].

#### Oral cephalosporins

Cefixime is an oral third-generation cephalosporin which inhibits bacterial cell wall synthesis by binding to one or more of the penicillin-binding proteins (PBPs), inhibiting cell wall synthesis. It is widely distributed throughout the body and reaches therapeutic concentration levels in most tissues and body fluids, with a time to peak serum concentration of 2–6 h and a half-life of 3–4 h [25]. Cefixime has been demonstrated to be effective (at 8 mg/kg/day in two divided doses) for shigellosis in adults and paediatric patients [26–29], although one study documented inferior efficacy to that of azithromycin [27]. Short (2-day) courses have been found to be as effective as 5-day courses [27,29]. Cefixime may be useful for paediatric patients when cephalosporin is necessary owing to high resistance to fluoroquinolones and β-lactams, and it can be taken orally. Cefixime is affordable and the suspension can be stored at room temperature [28]. Updated clinical trials to investigate this therapy as an alternative treatment option are urgently needed because previous randomised controlled trials investigating its efficacy are over a decade old.

No other antimicrobial agents were investigated in the research which met this review’s inclusion criteria.

### Synopsis of international guidelines

Four evidence-based international guidelines were reviewed (listed in order of most recently updated): the Infectious Diseases Society of America (IDSA) [30], Therapeutic Guidelines (Australia) [31], the American Academy of Pediatrics (AAP) [7] and BMJ Clinical Evidence [32]. Their recommendations are summarised in Table 4. Apart from the IDSA guidelines which are currently under review (last published in 2001), in line with the 2005 WHO guidelines [4], most international guidelines currently recommend fluoroquinolones as first-line therapy.

**Table 4.** Current international guidelines for the treatment of shigellosis.

Guideline	Last update	Recommendations
IDSA [30]	2001; update in progress	Based on A-1 level of evidence
		[Where: A = good evidence to support a recommendation for use; and I = Evidence from at least one properly randomised, controlled trial]
		Selective therapy should be instituted for shigellosis
		• TMP-SMZ 160 + 800 mg, respectively (paediatric dose 5 and 25 mg/kg, respectively) bd for 3/7 if susceptible)
		or
		• Fluoroquinolone: e.g. ciprofloxacin bd for 3/7 (paediatric dosing not listed); 300 mg ofloxacin; 400 mg norfloxacin; or 500 mg nalidixic acid 55 mg/kg/day for 5/7 • Ceftriaxone 100 mg/kg/day in 1 or 2 divided doses
Therapeutic Guidelines (Australia) [31]	2014	Selective therapy for:
		• Children < 6 years • Institutionalised populations or food handlers • MSM • Immunosuppressed • Patients with severe disease
		Empirical therapy (while awaiting local sensitivities):
		• Ciprofloxacin 500 mg (12.5 mg/kg up to 500 mg) PO bd for 5 days
		or
		• Norfloxacin 400 mg (10 mg/kg up to 400 mg) PO bd for 5 days
		or
		• TMP-SMZ 160 + 800 mg (4 + 20 mg/kg up to 160 + 800 mg) PO bd for 5 days
		Second-line therapy:
		• Azithromycin 500 mg (10 mg/kg up to 500 mg) PO on day 1, then 250 mg (5 mg/kg up to 250 mg) PO daily for a further 4 days
American Academy of Pediatrics [7]	2015	• Do not treat mild episodes • Selected therapy: for those with severe disease or immunosuppressed
		Empirical therapy (while awaiting culture/susceptibility results): any of (not hierarchical):
		• Ciprofloxacin 15 mg/kg bd for 3 days • Azithromycin 12 mg/kg on day 1; then 6 mg/kg on days 2–4 (total course: 4 days) • Parenteral ceftriaxone (50–75 mg/kg daily) for 2–5 days – for seriously ill patients
		The guidelines also note that oral cephalosporins (cefixime) have been used successfully in treating shigellosis in adults.
BMJ Clinical Evidence [32]	2016	Selective therapy for:
		• Malnourished, immunocompromised or elderly patients; food handlers, health care workers • Severe disease: defined as bloody diarrhoea with cramping while systemically unwell
		Empirical therapy (while awaiting local sensitivities):
		• Ciprofloxacin: 15 mg/kg (max 500 mg) PO bd
		or
		• Norfloxacin: 10 mg/kg (max 400 mg) PO bd
		Second-line therapy:
		• Ceftriaxone: 50–100 mg/kg IM once daily (adults: 1–2 g intramuscularly once daily)
		or
		• Azithromycin: 6–20 mg/kg PO once daily
		All therapies state ‘consult with a specialist for guidance on duration of treatment’
British National Formulary [33]	2016	Ciprofloxacin 20 mg/kg bd (higher dose than 15 mg/kg previously recommended)

Of note, comparison of international guidelines reveals differing dosage ranges for ciprofloxacin, from 12.5 mg/kg [31] to 20 mg/kg (BNF) [33], and the WHO 2005 guidelines list 15 mg/kg as the currently recommended dosage [4]. Ciprofloxacin has high oral bio-availability (approximately 70%) which is not influenced by the concurrent administration of feeds, and no substantial loss by first-pass metabolism. Maximum serum concentrations are attained 1 or 2 h after oral dosing, and its half-life is approximately 4 h in patients with normal renal function [32]. References supporting the efficacy of a higher dose range (20 mg/kg, above the currently recommended 15 mg/kg) were not found in the literature, and, owing to the associated risk of adverse events when combined with other CYP3A4 inhibitors (discussed below), there is no current evidence base to suggest that a higher dose of ciprofloxacin than currently recommended is warranted in shigellosis. Furthermore, higher minimum inhibitory concentrations (MIC) of fluoroquinolones requiring increased ciprofloxacin concentrations have not been found to be significantly associated with poorer clinical outcomes [30].

### Review of harms and toxicity – summary of evidence on safety

#### Adverse events

A 2010 systematic review of 1748 paediatric and adult patients [2] found no statistically significant differences in adverse events between patients taking fluoroquinolones, macrolides (including azithromycin) or β-lactams for shigellosis, concluding that all classes of currently available antibiotics for shigellosis are safe. The side effects of therapies currently recommended for shigellosis and those which may be considered in the future are highlighted in Table 5.

**Table 5.** Common adverse reactions to antibiotics currently indicated to treat shigellosis in children [7,33].

Antibiotic	Life-threatening	Mild adverse effects which may result in discontinuation of treatment	Other	Relevant interactions
Fluoroquinolones:CiprofloxacinNorfloxacinOfloxacin	Hypersensitivity reactions;	Dyspepsia, headache, diarrhoea, vomiting, hypotension	Tendonitis and tendon rupture; peripheral neuropathy.	All fluoroquinolones should be used with caution in patients receiving drugs known to prolong the QT interval
	Prolonged QT syndrome			The toxicity of fluoroquinolones is increased by the concurrent use of systemic steroidal medications
			A 2010 systematic review of ciprofloxacin safety in paediatrics concluded that although musculoskeletal adverse effects occur owing to ciprofloxacin use, these events are reversible [14]	
				Fluoroquinolones’ effects are reduced by the co-administration of iron- and zinc-containing products, of importance when zinc-containing products are used to treat diarrhoea in children.
				Fluoroquinolones cause additive toxicity with non-steroidal anti-inflammatory drugs (ibuprofen, meloxicam, naproxen)
Azithromycin	Hypersensitivity reactions;	Dyspepsia, flatulence, headache, disturbance in taste, anorexia	Malaise, paraesthesia	Macrolides use not advised with other drugs which prolong the QT interval, (including anti-malarial medications such as artemether-lumefantrine) owing to the risk of ventricular arrhythmias. However, azithromycin has been identified as a safer macrolide (in terms of its ability to prolong the QT interval) in this class of antibiotics.
	Prolonged QT syndrome			
				Plasma concentrations of azithromycin are increased by ritonavir
				Azithromycin in combination with rifabutin results in increased side-effects of rifabutin, including neutropenia
Ceftriaxone	Hypersensitivity reactions	Diarrhoea, headache, abdominal discomfort	Transient cholestatic jaundice owing to biliary sludge formation	Relevant interactions for all cephalosporins:
				Increased risk of nephrotoxicity when co-administered with aminoglycosides
				Enhance anticoagulant effect of coumarins
Cefixime	Hypersensitivity reactions; immune-mediated haemolytic anaemia	Flatulence, headache, abdominal pain, defaecation urgency, nausea, constipation, vomiting	Transient cholestatic jaundice owing to biliary sludge formation	As per ceftriaxone
Pivmecillinam	As with all penicillins: hypersensitivity reactions, serum-sickness-like reactions, anaphylaxis	Diarrhoea, joint pain, rashes, urticaria	Avoid use in acute porphyrias	Contra-indicated for concurrent use with sodium valproate

#### Possible cardiac side effects

Previous case reports of fluoroquinolones and macrolides have been associated with prolongation of the QT interval [34,35]. Independently, mild delays in ventricular repolarisation are clinically unnoticeable, although these antimicrobials may serve to amplify the risk of *torsades de pointes* (TdP), a potentially fatal polymorphic ventricular tachyarrhythmia which may present as sudden death (owing to ventricular tachycardia), syncope, palpitations, seizures or asymptomatically if the duration is short and it terminates spontaneously [35]. Of note, the current literature identifies this risk as requiring the presence of other risk factors, as highlighted in Table 6. The main reported risk factor for TdP is co-administration of other medications which are substrates and/or inhibitors of cytochrome P450 (CYP) enzymes, and the possibility of metabolic instability resulting from synergistic interactions with this enzyme. This risk is enhanced by individual allelic variations in CYP3A4, the most important enzyme in human drug metabolism. CYP3A4 is responsible for the biotransformation of approximately 60% of all oxidised drugs and allelic variations can result in patients being poor metabolisers of CYP3A4-inducing medications, resulting in reduced clearance of drug substrates and increasing exposure to the effects of toxicity [36].

**Table 6.** Risk factors for the development of *torsades de pointes* [34].

Risk factor	Examples
Genetic risk factors	Channelopathies
	CYP3A4 poor metaboliser
Underlying cardiac disease	Bradycardia
	Congestive cardiac failure
	Myocardial ischaemia
	Atrial fibrillation
Electrolyte derangements	Hypokalaemia
	Hypomagnesaemia
	Hypocalcaemia
Organ impairment, altering medication toxicity	Renal insufficiency
	Severe hepatic disease
Use of medication to increase QT liability	Concurrent CYP medications administered

Existing evidence suggests that the individual risk of cardiac arrhythmias secondary to these antimicrobials is minimal; yet, when combined with a genetic propensity to poor metabolism of CYP3A4-inducing medications and co-administration with other CYP potentiators, the risk may be magnified. How this might affect clinical practice, however, remains unclear. Another important risk factor to consider is the possibility of acute renal failure in the setting of severe dehydration secondary to shigellosis which could result in decreased clearance and enhanced toxic effects, increasing prolongation of the QT interval in a clinical setting.

#### Prolonged QT syndrome and azithromycin

As discussed, the predominantly reported risk of macrolide-associated TdP is the co-administration of other CYP3A4 inhibitors, resulting in increased drug toxicity. However, azithromycin has been identified as distinguishable from other macrolides as a group in terms of its cardiac toxicity as it minimally inhibits CYP3A4, resulting in a lack of appreciable interaction with other CYP3A4 substrates, and is classified as one of the safer macrolide antibiotics from a cardiac perspective [35,37]. In recent years, however, increasing attention has been paid to azithromycin’s risks following a documented increased risk of cardiac death in a cohort of 347,795 patients aged 30–74 years taking azithromycin [38]. The study found that patients taking 5 days of azithromycin, in comparison with taking no antibiotics, had a statistically significantly increased risk of cardiac death (hazard ratio 2.88, 95% CI 1.25–2.75, *p* < 0.0001) as well as of death from any cause (HR 1.85, 95% CI 1.25–2.75, *p* = 0.002). However, the risk was found to be most pronounced in patients with a high baseline risk of cardiovascular disease, and there was evidence of confounding by factors associated with both azithromycin use and risk of cardiovascular disease – namely a history of smoking, high body mass index, poor diet and low physical activity. At present, published case reports of an increased risk of sudden cardiac deaths in patients taking azithromycin are limited to adults [39,40].

#### Prolonged QT syndrome and fluoroquinolones

As with macrolides, there is interclass variability in the QT prolongation effect of fluoroquinolones. Ciprofloxacin’s inhibition of CYP1A2 has been described as ‘relatively inconsequential’ [35] and the US Food and Drug Administration (FDA)’s Adverse Event Reporting System (AERS) supports the notion of several causes of fluoroquinolone-associated TdP, usually in the context of co-administration with another QT-prolonging drugs, underlying cardiac disease, renal impairment and electrolyte anomaly.

#### Fluoroquinolone use and polyneuropathy

In 2013, the FDA issued a communiqué to specifically address the risk of peripheral neuropathy (PN) for all oral fluoroquinolones [41], mainly in response to case reports of this adverse event [42], in the absence of large epidemiological studies. The neurotoxic mechanism is thought to be through the inhibition of GABA-receptors which occurs within days of use and may be permanent. Between 1997 and 2012, the FDA’s AERS recorded 539 reports (1% of all submitted events for fluoroquinolones) pertaining to peripheral neuropathy. A review of these reports found that the majority of affected patients were female with a median age of 48 years (range 9–100) [43]. This evidence was further investigated by a 2014 pharmaco-epidemiological study which quantified the risk and demonstrated a relative risk of developing peripheral neuropathy with fluoroquinolone use of 2.07 (95% CI 1.56–2.74) [44]. With regard to ciprofloxacin specifically, the increased risk was quantified as RR 1.93 (95% CI 1.32–2.82), a small but appreciable increase. However, the research was based on a cohort of men with a mean age of 68 years, and it is difficult to extrapolate these data to the paediatric population. Because of the risk of permanent peripheral neuropathy resulting from fluoroquinolone use, the FDA’s most recent advice warns against the use of fluoroquinolone except when no other treatment is available [41]; yet, in the setting of global shigella spp. non-susceptibility, the public health benefits of treatment outweigh the small yet statistically significant risk of this adverse event.

#### Co-administration of azithromycin with artemisinin-based antimalarial drugs

Co-administration of macrolides or quinolones with other QT-prolonging agents could present a clinical problem, yet nearly all data examining the risk are for adults (often with pre-existing cardiac risks), and genetic differences between populations may limit interpretation. Azithromycin is a weak antimalarial that has been used in combination with several other anti-malarials or co-administered to treat non-malarial infections. Current evidence evaluating the cardiac risk of azithromycin co-adminsitered with chloroquine [45–47], artesunate/arthmeter [48,49] or piperaquine [50,51] does not identify an increased risk of cardiac instability in paediatric patients.

### Antimicrobial resistance patterns

Evidence suggests that antibiotic resistance is an increasing challenge in the therapeutic management of shigellosis, as recognised by the WHO prioritising ciprofloxacin-resistant shigella as a target of current international focus on antimicrobial resistance [52]. There are several mechanisms by which this may occur. In shigella spp., antimicrobial resistance is often owing to classes 1 and 2 integrons which contain resistance gene cassettes which are mobile and transferrable from one bacterium to another, providing a flexible way for bacteria to adapt to the environmental pressure caused by antibiotics. This may account for the dissemination of resistant genes and the emergence of multidrug-resistant strains, and explain why shigella resistance patterns vary worldwide – as the distribution of integrons varies according to the species and resistance phenotype (with. *S. sonnei* and *S. boydii* strains containing a single class 2 integron, while *S. flexneri* and *S. dysenteriae* carry a class 1 integron, often in combination with a class 2 integron which increases the propensity of dissemination of MDR strains of shigella) [53]. This underpins the importance of any antimicrobial resistance programme including surveillance to document changes in prevalent species in regions worldwide.

#### Resistance to fluoroquinolones

The primary target of fluoroquinolones is the DNA gyrase, a type II topoisomerase essential for DNA replication and transcription. Mutations in the gyrA gene have been shown to increase the MICs of fluoroquinolones for shigella spp. and other enterobacteriaceae, while plasmid-mediated quinolone resistance genes can also be acquired [54]. Of concern, complete ciprofloxacin resistance (MIC ≥ 4 mg/L) has recently been reported in domestic and imported *S. sonnei* isolates in the USA, Vietnam and elsewhere [55–57], and patients infected with fluoroquinolone-resistant shigella have a longer duration of diarrhoea than those with fluoroquinolone-susceptible strains [26]. Ciprofloxacin resistance must continue to be closely monitored and non-susceptibility should be reported when data are available.

#### Resistance to cephalosporins

Cephalosporin resistance is also of concern, arising from the production of plasmid-mediated β-lactamase [58]. Resistance to third-generation cephalosporins owing to the production of extended-spectrum β-lactamases (ESBLs) which confer resistance to all β-lactamases (except cephamycins and carbapenems) is increasingly prevalent and has been documented in recent laboratory analyses in Asia [59,60]. ESBL resistance in shigella spp. needs to be closely monitored in view of the necessity of treatment with expensive carbapenems, one of the last options for treating multi-resistant Gram-negative organisms, and there has been a considerable increase in the prevalence of ESBL resistance, including in shigella [53,59,60]. Thus, previous reports of the efficacy of ceftriaxone and cefixime might not reflect today’s susceptibility patterns; the use of these agents rapidly induces ESBLs and resistance to other antibiotic classes.

## Discussion

Shigella, a Gram-negative enterobacteriaceae, is responsible for 165 million diarrhoeal episodes each year, 99% of which occur in LMIC, and 69% in the paediatric population. With effective antibiotic therapy, there is clinical improvement within 48 h, diminishing the risk of mortality and decreasing transmission by eliminating shigella from the stool. The WHO 2005 Guidelines for the Control of Shigellosis, Including Epidemics due to Shigella Dysenteriae Type 1 listed the fluoroquinolone ciprofloxacin (15 mg/kg orally twice daily for 3 days) as first-line treatment for shigellosis in children, and (more expensive and less available) pivmecillinam (amdinocillin pivoxil) and (parenteral) ceftriaxone were listed as second-line therapy when local strains were known to be resistant to ciprofloxacin. The macrolide azithromycin was listed as a second-line therapy for adults.

There is a lack of current research on the clinical treatment of shigellosis in paediatric or adult patients, despite rising antimicrobial non-susceptibility rates worldwide. In particular, there is a lack of research assessing the non-susceptibility of community-acquired strains; almost all published research pertains to microbiological data from hospital-based settings. Research investigating non-susceptibility of community-acquired shigellosis is urgently required. A large proportion of the current evidence is based on *in vitro* studies which do not necessarily correspond with clinical outcomes, and studies of clinical efficacy do not evaluate individual drugs.

A number of international guidelines currently list azithromycin as a first- and second-line therapy for shigellosis in children. While there are no published trials comparing the efficacy of azithromycin with that of ciprofloxacin for shigellosis in children, previous trials in adults have demonstrated similar efficacy and higher *in vitro* susceptibility; however, reports of azithromycin-resistant strains are increasing. In areas where ciprofloxacin-resistance is evident, azithromycin is an appropriate second-line alternative therapy owing to its oral administration and affordability. Safety concerns (the risk of polyneuropathy in ciprofloxacin use and prolonged QT syndrome secondary to azithromycin) are based mainly on retrospective studies in adults and results cannot necessarily be extrapolated to the paediatric population, although care should be taken when co-administering with other CYP450 inducing medications.

Prior research has investigated the antibiotics currently recommended by WHO together rather than assessing individual therapies. Research investigating non-susceptibility of ahigellosis – particularly community-acquired strains – is urgently required, and *in vitro* non-susceptibility studies need to be correlated with clinical outcomes. Further randomised controlled trials adhering to CONSORT guidelines are required to guide future treatment options for shigellosis, especially in populations at risk of high case fatality (such as malnourished or HIV-positive children). The efficacy of oral cephalosporins (cefixime) for shigellosis should be a priority for research as it is affordable and easily administered compared with (the currently recommended) parenteral ceftriaxone. Specifically, a large randomised controlled trial in children in the Asia–Africa region should compare the efficacy of ciprofloxacin, azithromycin and cefixime as the first-line treatment of shigellosis in children, including the assessment of MICs in relation to treatment outcomes and addressing the risks of exacerbating resistance in other intestinal bacteria, especially ESBL-producing enterobacteriaceae.

In conclusion, current WHO guidelines supporting the use of fluoroquinolones (first-line), β-lactams (second-line) and cephalosporins (second-line) accord with currently available evidence and other international guidelines, and there is no strong evidence to change this guidance. Azithromycin may be considered as an appropriate second-line therapy in regions with known high rates of ciprofloxacin non-susceptibility. Cefixime is also a reasonable alternative, although its use must be balanced against the risk of increasing antimicrobial resistance and the spread of ESBL.

## Notes on contributors

***Phoebe C. M. Williams***, MBBS (Hons.), received her medical degree from The University of Sydney and a Masters in Global Health Science from The University of Oxford. She is a paediatric registrar and dual trainee in Infectious Diseases at Sydney Children’s Hospital, Australia. She is a DPhil candidate through The University of Oxford, with her research focusing on antimicrobial resistance in paediatric patients.

***James A. Berkley*** FRCPCH, MD is a professor of Paediatric Infectious Diseases at The University of Oxford based at the KEMRI-Welcome Trust Research Programme in Kilifi, Kenya. He is the principal investigator of the CHAIN network with a research focus on serious infection and survival in highly vulnerable groups of infants and children.

## Funding

This work was supported by The World Health Organization, The Nuffield Department of Medicine (University of Oxford), The General Sir John Monash Foundation, The Wellcome Trust and the Bill and Melinda Gates Foundation.

## Disclosure statement

No potential conflict of interest was reported by the authors.

## Appendix 1

**Table:**

Author	Year	Title	Setting	Population	Method	Findings	Level of evidence
Das et al. [12]	2013	Antibiotics for the treatment of cholera, ahigella and xryptosporidium in children	Systematic review and meta-analysis (international)	Children <16 years	The CHERG standard rules were applied to determine the final effect of treatment with antibiotics on diarrhoea morbidity and mortality	Using clinical failure rates as a proxy for shigella deaths (as there was no data on mortality), the authors propose that treating shigella dysentery with antibiotics results in an 82% (67–99%) *reduction in diarrhoea mortality* from shigella.	A
				48 papers relevant to Ahigella were included, 47 of which were from developing country settings			
					Studies were included if they reported the effect of antibiotics on morbidity and mortality associated with diarrhoea owing to cholera, shigella and cryptosporidiosis in children, as observed by clinical and bacteriological failure and mortality. No studies compared antibiotics with placebo or a control group, so studies with an antibiotic comparison group were also included. Only studies with a confirmed diagnosis of the respective infection and on immunocompetent patients were included		
				All studies were hospital-based			
						*Bacteriological failure*: Antibiotics (including ciprofloxacin, pivmecillinam and ceftriaxone) successfully clear shigella pathogens in 96% (88–99%) of cases	
						The overall conclusion is that there is strong evidence of effectiveness against serious mortality and morbidity	
						All antimicrobials were analysed together	
Gu et al. [8]	2012	Comparison of the prevalence and changing resistance to nalidixic acid and ciprofloxacin of Ahigella between Europe–America and Asia–Africa from 1998 to 2009.	Systematic review comparing Africa-Asia region with Europe–America	26,877 specimens from adults and children in LMIC and HIC	SR for articles published during 1998–2011 using search strategy ‘bacterial surveillance’ OR ‘antimicrobial resistance’ OR ‘bacterial resistance’ AND ‘shigella’	The predominant species isolated was *S. sonnei*, representing 8900 (56.6%) of total isolates, followed by *S. flexneri* (5749 isolates, 36.5%), *S. boydii* (601 isolates, 3.8%) and *S. dysenteriae* (481 isolates, 3.1%)	A
					102 studies met inclusion criteria, LMIC and HIC all included	Quinolone resistance in Asia–Africa:	
						• NALIDIXIC ACID: Resistance to nalidixic acid increased gradually from 12.1% (95% CI 4.3–23.2) from 1998–2000, to 64.5% (13.8–99.3) in 2007–2009; a 5.3-fold increase in resistance rates • CIPROFLOXACIN: Resistance to ciprofloxacin increased from 0.6% (0.2–1.3) in 1998–2000 to 29.1% (0.9–74.8) in 2007–2009; a 49-fold increase in resistance over 12 years • Overall rates are much higher than those documented in:	
						Quinolone resistance in Europe–America:	
						• NALIDIXIC ACID: 1.3% (0.6–2.1) 1998–2000 to 2.1% (1.3–3.0) in 2007–2009 • CIPROFLOXACIN: 0.0% (1998–2000) to 0.6% (0.2–1.2) in 2007–2009 • i.e. SIGNIFICANTLY lower than resistance rate increases in Africa–Asia • *S. flexneri* showed higher resistance rates than *S. sonnei* to ciprofloxacin, with a general upward trend in resistance over time internationally • In Asia–Africa, the resistance patterns differed – *S. sonnei* appeared to be more resistant than *S. flexneri* to nalidixic acid • Resistance rates to quinolones were much greater in children than in adults, with the respective rates being 33.05% (23.9–42.8) *vs* 14.3% (8.30–21.7) for nalidixic acid, and 7.5% (4.3–11.5) *vs* 3.6% (2.2–5.3) for ciprofloxacin	
					In all the studies collected, a total of 26,877 isolates were positive for shigella, 15,731 of which had information about prevalence of subtypes		
						Owing to widespread use of nalidixic acid as the first-line agent for empirical treatment of infectious diarrhoea, resistance to nalidixic acid in Asian–African countries increased to 64.5% (95% CI 13.8–99.3) in 2007–2009. Thus, this drug should no longer be considered appropriate empirical therapy	
						Progressively increasing resistance to ciprofloxacin is still a serious cause of concern and several studies have emphasised that most nalidixic acid-resistant strains exhibit some degree of cross-resistance to ciprofloxacin	
Gu et al. [17]	2013	Prevalence and trends of aminoglycoside resistance in shigella worldwide, 1999–2010	Systematic review and meta-analysis evaluating aminoglycoside resistance in shigella, Asia–Africa *vs* Europe–America	Adults and children	3176 publications were retrieved from MEDLINE and EMBASE reported from 1999 to 2012, 68of which met the inclusion criteria	• The summarised prevalence of gentamicin, kanamycin and amikacin resistance was found to be 3.95% (95% CI 3.59–4.22) (*n/N* = 937/14,059), 6.88% (6.36–7.43) • (*n/N* = 1106/8,647) and 1.29% (0.97–1.68) • (*n/N* = 432/8614), respectively. Importantly, evident heterogeneity was observed (p < 0.001). • The most common drug resistance was observed for kanamycin. Re. kanamycin resistance, the highest drug resistance rate by geographic areas was in Asia with a prevalence of 16.78% (7.58–28.71). • Similarly, the most common resistance was observed for 2005–2007 and S. flexneri with a summarised combined prevalence of 12.05% (11.18–14.21) and 9.25% (7.69–10.96), respectively. • A lower prevalence of gentamicin resistance was found in European–American countries at 0.68% (0.39–1.05). • After analysing the study data on years, we observed a minimal change in the resistance prevalence of gentamicin, from 0.25% (0.04–0.64) to 0.84% (0.08–2.40) in European–American countries, in contrast to data in Asian–African countries, which fluctuated from 6.05% (1.18–14.28) to 20.83% (12.67–30.40). • It is worth noting that the resistance prevalence of gentamicin increased annually in Asian–African countries, while the resistance prevalence decreased year by year in European–American countries: The prevalence of gentamicin resistance in Asian–African countries increased sharply from 14.00% (3.97–28.85) in 2002–2004 to 20.96% (3.37–48.11) in 1999–2001 and to 32.40% (17.87–48.91) in 2005–2007. Data for Asian–African regions from 2008–2010 were not found. • The changes in kanamycin resistance in European–American countries were minimal; in fact, the resistance prevalence decreased annually. • In European–American regions, a lower amikacin resistance was also found during the 12-year study period (decreased from 0.28% (0.00–1.08) to 0.05% (0.04–0.40). The highest resistance of shigella isolates to amikacin was only 0.28% (0.00–1.08). • The prevalence of amikacin resistance remarkably increased from 6.39% (1.40–14.63) to 48.06% (34.57–61.65) in Asian–African countries. • In the paediatric age group, resistance of shigella to gentamicin was higher than in adults [5.93% (3.97–8.23) and 18.34% (9.81–28.76)]. • Kanamycin resistance in the paediatric age group was significantly higher than in the adults which showed 70.72% (33.95–96.25) *vs* 5.40% (1.87–10.62) for kanamycin. • Similarly, there was greater resistance to amikacin in the paediatric group than in the adult group [8.43% (3.26–15.71) *vs* 2.23% (0.81–4.35)].	A
Gu et al. [15]	2015	Comparison of resistance to 3rd–generation cephalosporins in shigella between Europe–America and Asia–Africa from 1999–2012	Europe–America and Asia–Africa	Adults and children	A systematic review was conducted to compare resistance to 3rd-generation cephalosporins in shigella strains between Europe–America and Asia–Africa from 1998 to 2012	• In Asia–Africa, the prevalence of resistance of total and different subtypes to ceftriaxone, cefotaxime and ceftazidime increased markedly over the study period, with a total prevalence of resistance up to 14·2% [95% confidence interval (CI) 3·9–29·4], 22·6% (95% CI 4·8–48·6) and 6·2% (95% CI 3·8–9·1) during 2010–2012, respectively. • By contrast, resistance rates to these TGCs in Europe–America remained relatively low – <1% during the 15 years • A noticeable finding was that certain countries in Europe–America and Asia–Africa had a rapid rising trend in the prevalence of resistance of *S. sonnei* which even outnumbered *S. flexneri* in some periods • Comparison between countries showed that currently the most serious problem concerning resistance to these TGCs was in Vietnam especially for ceftriaxone, in China especially for cefotaxime and in Iran especially for ceftazidime. • Changes in the prevalent serogroups and resistance patterns in antimicrobial susceptibilities in shigella are posing major difficulties in determining an appropriate drug for the treatment of shigellosis • Based on our meta-analyses, two main recommendations can be given for empirical antibiotic therapy. First, the current situation in Europe–America supports the use of ceftriaxone and cefotaxime for treating shigellosis according to the relatively lower prevalence of resistance to the study drugs (although a mild upward trend should be noticed). To some extent, data suggest that ceftriaxone and cefotaxime may not be appropriate for shigellosis in Asia–Africa.	A
					Only high quality studies were included for analysis (defined as prospective cohort or retrospective consecutive cohort studies where susceptibility tests were conducted according to CLSI guidelines with external quality control). 104 articles met these inclusion criteria		
Traa et al. [14]	2010	Antibiotics for the treatment of dysentery in children.	Systematic review	Children aged <16 years	Systematic review investigating the effect of ciprofloxacin, ceftriaxone and pivmecillinam for dysentery in children in developing countries; CHERG Standard Rules were applied	• Treatment with ciprofloxacin, ceftriaxone or pivmecillinam resulted in a clinical *failure* rate of 0.1% (95% CI – 0.2 to 0.5 – *not significant*) • Treatment with ciprofloxacin, ceftriaxone or pivmecillinam resulted in a cure rate of >99% while assessing clinical failure, bacteriological failure and bacteriological relapse • Therefore, the antibiotics recommended by WHO are effective in reducing the clinical and bacteriological signs and symptoms of dysentery and thus can be expected to decrease diarrhoea mortality attributable to dysentery • NB: The methods of susceptibility testing were not discussed and treatment benefit was summarised for all therapies together	B
					8 papers were selected for abstraction; 4 reported on bacteriological failure and 5 on bacteriological relapse		
					All studies were RCTs judged to be of good quality		
			Developing countries				
			All studies were conducted in clinical or hospital settings				
von Seidlein et al. [9]	2006	A multicentre study of shigella diarrhoea in six Asian countries: disease burden, clinical manifestations, and microbiology	Surveillance of 600,000 persons of all ages	Bangladesh, China, Pakistan, Indonesia, Vietnam and Thailand	Shigella was isolated from 2927 (5%) of 56,958 diarrhoea episodes detected between 2000 and 2004	• The overall incidence of treated shigellosis was 2.1 episodes per 1000 residents per year in all ages and 13.2/1000/y in children under 60 months. • *S. flexneri* was the most frequently isolated shigella species (1976/2927 [68%]) in all sites except Thailand, where *S. sonnei* was most frequently detected [124/146, 85%). • The majority of *S. flexneri* isolates in each site were resistant to amoxicillin and cotrimoxazole. Ciprofloxacin-resistant *S. flexneri* isolates were identified in China (18/305, 6%), Pakistan (8/242, 3%) and Vietnam (5/282, 2%)	B
					Swabs were inoculated onto MacConkey agar and salmonella-shigella agar and incubated overnight then checked for non-lactose fermenting enteropathogens		
					Antimicrobial sensitivity testing against ampicillin, cotrimoxazole, nalidixic acid and ciprofloxacin was performed by disk diffusion following CLS methods, and subject to external laboratory validation		
Vinh et al. [18]	2011	A multi-centre randomized trial to assess the efficacy of gatifloxacin vs ciprofloxacin for the treatment of shigellosis in Vietnamese children	Vietnam	494 children <15 years admitted to a paediatric ward with a history of passing bloody or mucoid stools, with or without abdominal pain, tenesmus or fever for <72 hours prior to admission	Randomised, open-label, controlled trial with two parallel arms at two hospitals in southern Vietnam	• We could not demonstrate superiority of gatifloxacin and observed similar clinical failure rates in both groups (gatifloxacin 12.0% and ciprofloxacin 11.0%, *p*=0.72). • The median (inter-quartile range) time from illness onset to cessation of all symptoms was 95 (66–126) hours for gatifloxacin recipients and 93 (68–120) hours for the ciprofloxacin recipients [HR (95% CI) 0.98 (0.82–1.17), *p*=0.83]. • Gatifloxacin showed a similar efficacy of both drugs in the treatment of childhood dysentery, including those with a stool culture-confirmed shigella infection. • However, gatifloxacin has a longer half-life than ciprofloxacin and the once-a-day administration may be considered more convenient than the twice-a-day regimen of ciprofloxacin. • Data show similar overall risks of treatment failure in the 2 treatment groups (11% in the ciprofloxacin group *vs* 12%) • The most commonly isolated shigella species here was *S. sonnei* • *S. dysenteriae* causes a considerably more severe syndrome than *S. sonnei* which is largely associated with the secretion of shiga toxin • These data suggest that whilst there has been a notable increase in MIC to nalidixic acid in shigella in Vietnam over the last 10 years, it may not yet be substantial enough to hinder the bactericidal effect of ciprofloxacin *in vivo*. • A similar effect of both antimicrobial agents, despite gatifloxacin having greater in vivo activity, supports the theory of a less severe infection which may not in all cases require an antimicrobial for the cessation of symptoms. Alternatively, shigella may respond in an atypical manner to gatifloxacin with respect to other gram-negative organisms, and mutations in the gyrA and parC gene may have a greater effect in reducing the potency of the antimicrobial agent. • We conclude that in Vietnam, where nalidixic acid resistant Shigellae are highly prevalent, ciprofloxacin and gatifloxacin are similarly effective for the treatment of acute shigellosis	B
				EXCLUSION CRITERIA = any prior treatment with a fluoroquinolone during the current bout of disease, or children with a trophozoite or *Entamoeba histolytica* in their stool on microscopic examination			
					The study was designed as a superiority trial and children with dysentery meeting the inclusion criteria were invited to participate		
					Participants received either gatifloxacin (10 mg/kg/day) in a single daily dose for 3 days or ciprofloxacin (30 mg/kg/day) in two divided doses for 3 days		
					The primary outcome measure was treatment failure; secondary outcome measures were time to the cessation of individual symptoms		
					494 patients were randomised to receive either gatifloxacin (*n* = 249) or ciprofloxacin (*n* = 245), of 107 of whom had a positive shigella stool culture		
Thompson et al. [21]	2016	Clinical implications of reduced susceptibility to fluoroquinolones in paediatric *Shigella sonnei* and *Shigella flexneri* infections	Vietnam	490 paediatric patients <15 years admitted to tertiary units in Vietnam	Clinical information and bacterial isolates were derived from a randomised controlled trial comparing gatifloxacin with ciprofloxacin (above, Vinh et al.) for paediatric shigellosis.	• *Shigella flexneri* patients treated with gatifloxacin had significantly worse outcomes than those treated with ciprofloxacin. • However, the MICs of fluoroquinolones were not significantly associated with poorer outcome. • The presence of S83L and A87T mutations in the gyrA gene significantly increased MICs of fluoroquinolones. • Elevated MICs and the presence of the qnrS gene allowed shigella to replicate efficiently *in vitro* in high concentrations of ciprofloxacin. • *Conclusions:* We found that below the CLSI breakpoint, there was no association between MIC and clinical outcome in paediatric shigellosis infections. However, *S. flexneri* patients had worse clinical outcomes when treated with gatifloxacin in this study regardless of MIC. • Shigella harbouring the qnrS gene are able to replicate efficiently in high concentrations of ciprofloxacin and we hypothesise that such strains possess a competitive advantage against fluoroquinolone-susceptible strains owing to enhanced shedding and transmission. • NOTE: Data collected between 2006 and 2009	C
					Time-kill experiments were performed to evaluate the impact of MIC on *in-vitro* growth of shigella, and Cox regression modelling was used to compare clinical outcome between treatments and shigella species		
					Patients were excluded if they had		
					received any fluoroquinolones within the time of this bacterial illness.		
					-Stool samples were collected on admission and standard microbiological techniques were employed to identify shigella and salmonella isolates-Antimicrobial susceptibility testing was performed by disc diffusion following methods prescribed by the CLSI-MICs were calculated by Etest as per the manufacturer’s instructions (AB Biodisk, Sweden). -Strains identified as resistant to ceftriaxone were subjected to further phenotypic tests to confirm ESBL production using discs containing only cefotaxime (30 mg) and both cefotaxime and ceftazidime combined with clavulanic acid (10 mg), according to current CLSI guidelines		
Christopher et al. [2]	2010	Antibiotic therapy for shigella dysentery	Systematic review (international)	16 RCTs met the inclusion criteria, which totalled 1748 children and adults based on clinical symptoms of dysentery prior to bacteriological confirmation	Of the 16 RCTs included, this was composed of 2 RCTs comparing antibiotics and placebo *vs* no drug; and 14 RCTs comparing effectiveness of different antibiotic regimens for treatment of shigella	There was insufficient evidence to consider any class of antibiotic superior in efficacy in treating shigella dysentery, but heterogeneity for some comparison limits confidence in the results	C
						There were no statistically significant differences in adverse events between participants taking macrolides, β-lactams or fluoroquinolones, leading the authors to conclude that all antibiotics were safe	
						There was inadequate evidence regarding the role of antibiotics in prevention of complications]	
						Conclusion: Low- to moderate-quality evidence that antibiotic therapy significantly reduces the number of children with dysentery on follow-up compared with no antibiotic	
					All RCTs were low- to moderate-quality evidence: of the 16 trials, 7 were at risk of bias owing to inadequate allocation concealment, and 12 owing to incomplete reporting of outcome data		
					Limited data from one 3-armed trial of people with moderately severe illness suggest that antibiotics reduce the episodes of diarrhoea at follow-up		
					Many RCTs included in the review were conducted before 1990 and included Abx no longer used owing to high resistance (cotrimoxazole, ampicillin, nalidixic acid)		
					Reviewed both developed and developing countries, limiting generalisability of findings to developing country settings		

## References

[1] Ashkenazi S Shigella infections in children: new insights. Semin Pediatr Infect Dis. 2004;15:246–252. 15494948 10.1053/j.spid.2004.07.005

[2] Christopher PRH, David KV, John SM, et al Antibiotic therapy for Shigella dysentery. Cochrane Database Sys Rev. 2010;8:CD006784 DOI:10.1002/14651858.CD006784.pub4. PMC6532574 20687081

[3] Liu J, Platts-Mills J, Juma J, et al Use of quantitative molecular diagnostic methods to identify causes of diarrhoea in children: a reanalysis of the GEMS case-control study. Lancet. 2016;388:1291–1301. 27673470 10.1016/S0140-6736(16)31529-X PMC5471845

[4] World Health Organization Guidelines for the control of shigellosis, including epidemics due to *Shigella dysenteriae type* 1. 2005 Available from: http://www.who.int/cholera/publications/shigellosis/en/

[5] Taylor M Enterohaemorrhagic *Escherichia coli* and *Shigella dysenteriae* type 1-induced haemolytic uraemic syndrome. Paediatr Nephrol. 2008;23:1425–1431. 10.1007/s00467-008-0820-3 PMC2459235 18493800

[6] Kotloff K, Winickoff JP, Ivanoff B, et al Global burden of Shigella infections: implications for vaccine development and implementation of control strategies. Bull WHO. 1999;77:651–666. 10516787 PMC2557719

[7] American Academy of Pediatrics Shigellosis In: Kimberlin M, Jackson M, Long S, et al, editors. Red Book: 2015. report of the committee on infectious diseases, 30th ed. Elk Grove Village (IL): American Academy of Pediatrics, 2015; p. 706–709.

[8] Gu B, Pan S, Zhuang L, et al Comparison of the prevalence and changing resistance to nalidixic acid and ciprofloxacin of Shigella between Europe–America and Asia–Africa from 1998 to 2009. Int J Antimicrob Agents. 2012;40:9–17. 22483324 10.1016/j.ijantimicag.2012.02.005

[9] von Seidlein KDR, Ali M, Lee H, et al A multicentre study of Shigella Diarrhoea in six Asian countries: disease burden, clinical manifestations, and microbiology. PLoS Med. 2006;3:e353. 16968124 10.1371/journal.pmed.0030353 PMC1564174

[10] World Health Organization Pocket book of hospital care for children. 2nd ed Geneva: WHO; 2013. 24006557

[11] Balshem H, Helfand M, Schünemann H, et al Grade guidelines 3: rating the quality of the evidence – introduction. J Clin Epidemiol. 2011;64:401–406. 21208779 10.1016/j.jclinepi.2010.07.015

[12] Das A, Salam R, Bhutta Z, et al Antibiotics for the treatment of cholera, shigella and cryptosporidium in children. BMC Public Health. 2013;Suppl. 3:S3–S10. 10.1186/1471-2458-13-S3-S10 PMC3847295 24564492

[13] Walker C, Bryce J, Bahl R, et al Standards for CHERG reviews of intervention effects on child survival. Int J Epidemiol. 2010;39:i21–i31. 20348122 10.1093/ije/dyq036 PMC2845875

[14] Traa C, Walker C, Munos M, et al Antibiotics for the treatment of dysentery in children. Int J Epidemiol. 2010;39(Supplement 1):i70–i74. 20348130 10.1093/ije/dyq024 PMC2845863

[15] Gu B, Zhou M, Ke X, et al Comparison of resistance to third-generation cephalosporins in Shigella between Europe–America and Asia–Africa from 1998 to 2012. Epidemiol Infect. 2015;143:2687–2699. 25553947 10.1017/S0950268814003446 PMC9151070

[16] Gu B, Zhou M, Ke X, et al Prevalence and trends of aminoglycoside resistance in Shigella worldwide, 1999–2010. J Biomed Res. 2013;27:103–115. 23554801 10.7555/JBR.27.20120125 PMC3602868

[17] Vinh V, Campbell J, Hoang N, et al A multi-center randomized trial to assess the efficacy of gatifloxacin versus ciprofloxacin for the treatment of shigellosis in vietnamese children. PLoS Negl Trop Dis. 2011;5:e1264. 21829747 10.1371/journal.pntd.0001264 PMC3149021

[18] Shanks B, Aleman G, Oundo J, et al Single dose of azithromycin or three‐day course of ciprofloxacin as therapy for epidemic dysentery in Kenya. Clin Infect Dis. 1999;29:942–943. 10589921 10.1086/520469

[19] Das AS, Ferdous F, Farzana F, et al Etiological diversity of diarrhoeal disease in Bangladesh. J Infect Dev Ctries. 2013;7:900–909. 24334935 10.3855/jidc.3003

[20] Das SK, Klontz EH, Azmi IJ, et al Characteristics of multidrug resistant Shigella and *Vibrio cholerae* 01 infections in patients treated at an urban and a rural hospital in Bangladesh. ISRN Microbiol. 2013;213915. 10.1155/2013/213915 PMC3881581 24455398

[21] Thompson N, Vinh P, Duc A, et al Clinical implications of reduced susceptibility to fluoroquinolones in paediatric *Shigella sonnei* and *Shigella flexneri* infections. J Antimicrob Chemother. 2016;71:807–815. 26679253 10.1093/jac/dkv400 PMC4743702

[22] Heiman J, Sjolund-Karlsson M, Bowen A, et al Shigellosis with decreased susceptibility to azithromycin. Paediatr Infect Dis J. 2016;33:1204–1205. 10.1097/INF.0000000000000397 PMC4700834 25361413

[23] Centers for Disease Control and Prevention Ciprofloxacin- and azithromycin-nonsusceptible Shigellosis in the United States. CDC Health Alert Network; 2015 Available from: https://emergency.cdc.gov/han/han00379.asp

[24] Howie RL, Bowen A, Barzilay EJ, et al Reduced azithromycin susceptibility in *Shigella sonnei,* United States. Microbiol Drug Resist. 2010;16:245–248. 10.1089/mdr.2010.0028 20624094

[25] Guay R, Harding G, Meatherall D Pharmacokinetics of cefixime in healthy subjects and patients with renal insufficiency. Antimicrob Agents Chemother. 1986;30:485–490. 3777912 10.1128/aac.30.3.485 PMC180585

[26] Ashkenazi S, Waisman Y, Rachmel A, et al A randomised, double-blind study comparing cefixime and trimethoprim-sulfamethoxazole in the treatment of childhood Shigellosis. J Pediatr. 1993;123:817–821. 8229498 10.1016/s0022-3476(05)80867-4

[27] Basualdo W, Arbo A Randomized comparison of azithromycin versus cefixime for treatment of shigellosis in children. Paediatr Infect Dis. 2003;22:374–373. 12712971

[28] Girgis N, Hammad O, Farid Z, et al Comparison of the efficacy, safety and cost of cefixime, ceftriaxone and aztreonam in the treatment of multidrug-resistant *Salmonella typhi* septicemia in children. Pediatr Infect Dis J. 1995;14:603–605. 7567290 10.1097/00006454-199507000-00010

[29] Martin R, Maffei F, Tritt J, et al Treatment of shigellosis with cefixime: two days vs. five days. Paediatr. Infect. Dis. 2000;19:522–526. 10.1097/00006454-200006000-00006 10877166

[30] Guerrant T, Steiner T, Thielman N, et al Practice guidelines for the management of infectious diarrhea. Clin Infect Dis. 2001;32:331–350. 11170940 10.1086/318514

[31] Shigella [revised November 2014], eTG complete [Internet]. Melbourne: Therapeutic Guidelines Limited; 2015.

[32] Keshav A, Acheson D, Allerberger F, et al BMJ best practice: Shigella infection. 2016 [Online]. Available from: http://bestpractice.bmj.com.acs.hcn.com.au/best-practice/monograph-pdf/1174.pdf

[33] British National Formulary for Children BMJ Publishing Group. 2016 Available from: https://www.medicinescomplete.com.acs.hcn.com.au/about/publications.htm

[34] Hancox M, Vieweg V, Crouse E, et al Azithromycin, cardiovascular risks, QTc interval prolongation, torsade de pointes, and regulatory issues: a narrative review based on the study of case reports. Ther Adv Infec Dis. 2013;1:155–165. 25165550 10.1177/2049936113501816 PMC4040726

[35] Owens RC, Nolin T Antimicrobial-associated QT interval prolongation: pointes of interest. Clin Infect Dis. 2006;43:1603–1611. 17109296 10.1086/508873

[36] Dresser GK, Bailey DG, Spence J Pharmacokinetic-pharmacodynamic consequences and clinical relevance of cytochrome P450 3A4 inhibition. Clin Pharmacokinet. 2000;38:41–57. 10668858 10.2165/00003088-200038010-00003

[37] Ohara Y, Watanabe Y, Cao X, et al Azithromycin can prolong QT interval and suppress ventricular contraction, but wi not induce Torsade de Pointes. Cardiovasc Toxicol. 2014;15:232–240. 10.1007/s12012-014-9289-4 25367413

[38] Ray W, Murray K, Hall K, et al Azithromycin and the risk of cardiovascular death. N Engl J Med. 2012;366:1881–1890. 22591294 10.1056/NEJMoa1003833 PMC3374857

[39] Samarendra P, Evans S, Sacchi TJ, et al QT prolongation associated with azithromycin/amiodarone combination. Clin Electrophysiol. 2001;24:1572–1574. 10.1046/j.1460-9592.2001.01572.x 11707055

[40] Howard P Azithromycin-induced proarrhythmia and cardiovascular death. Ann Pharmacol. 2013;47:1547–1551. 10.1177/1060028013504905 24285766

[41] Food and Drug Safety Administration Drug Safety Communication. Azithromycin (Zithromax or Zmax) and the rik of potentially fatal heart rhythms. 2013 [Online]. Available from: https://www.fda.gov/Drugs/DrugSafety/ucm341822.htm

[42] Etminan J, Samii A, Brophy M Oral fluoroquinolone use and risk of peripheral neuropathy. Neurology. 2014;83:1261–1271. 25150290 10.1212/WNL.0000000000000846

[43] Food and Drug Administration FDA updates warnings for fluoroquinolone antibiotics. 2016 [Online]. Available from: http://www.fda.gov/NewsEvents/Newsroom/PressAnnouncements/ucm513183.htm.

[44] Ali A Peripheral neuropathy and Guillain-Barré syndrome risks associated with exposure to systemic fluoroquinolones: a pharmacovigilance analysis. Ann Epidemiol. 2014;24:279–285. 24472364 10.1016/j.annepidem.2013.12.009

[45] Chandra P, Sagara I, Sie A, et al Comparison of azithromycin plus chloroquine versus artemether-lumefantrine for the treatment of uncomplicated *Plasmodium falciparum* malaria in children in Africa: a randomized, open-label study. Malaria J. 2015;14:108–116. 10.1186/s12936-015-0620-8 PMC4358906 25881046

[46] Kimani K, Kamiza S, Duparc S, et al Efficacy and safety of azithromycin-chloroquine versus sulfadoxine-pyrimethamine for intermittent preventive treatment of plasmodium falciparum malaria infection in pregnant women in Africa: an open-label, randomized trial. PLoS One. 2016;21:e0157045. 10.1371/journal.pone.0157045 PMC4915657 27326859

[47] Fossa T, Duncan J, Deng S, et al Azithromycin/chloroquine combination does not increase cardiac instability despite an increase in monophasic action potential duration in the anesthetized guinea pig. Am J Trop Med Hyg. 2007;77:929–938. 17984356

[48] Egunsola K, Oshikoya O Comparative safety of artemether-lumefantrine and other artemisinin-based combinations in children: a systematic review. Malaria J. 2013;12:385–401. 10.1186/1475-2875-12-385 PMC3818443 24175945

[49] Sykes I, Mtove G, Mandea V, et al Azithromycin plus artesunate versus artemether‐lumefantrine for treatment of uncomplicated malaria in Tanzanian children: a randomized, controlled trial. Clin Infect Dis. 2009;49:1195–1201. 19769536 10.1086/605635

[50] Baiden A, Oduro A, Halidou T, et al Prospective observational study to evaluate the clinical safety of the fixed-dose artemisinin-based combination Eurartesim® (dihydroartemisinin/piperaquine), in public health facilities in Burkina Faso, Mozambique, Ghana, and Tanzania. Malaria J. 2015;14:160. doi:10.1186/s12936-015-0664-9. PMC4405867 25885858

[51] Manning P, Lon C, Spring M, et al Randomized, double-blind, placebo-controlled clinical trial of a two-day regimen of dihydroartemisinin-piperaquine for malaria prevention halted for concern over prolonged corrected QT interval. Antimicrob Agents Chemother. 2014;58:6056–6057. 25092702 10.1128/AAC.02667-14 PMC4187937

[52] World Health Organization Antimicrobial Resistance Global Report on Surveillance. 2014 [Online]. Available from: http://apps.who.int/iris/bitstream/10665/112642/1/9789241564748_eng.pdf.

[53] Zhang CL, Liu QZ, Wang J, et al Epidemic and virulence characteristic of Shigella spp. with extended-spectrum cephalosporin resistance in Xiaoshan District, Hangzhou, China. BMC Infect. Dis. 2014;14:107 DOI:10.1186/1471-2334-14-260. 24886028 PMC4229937

[54] Bowen A, Hoover J, Hurd C Importation and domestic transmission of *Shigella sonnei* resistant to ciprofloxacin – United States, May 2014 – February 2015. Morbid Mortal Wkly Rep. 2015;64:318–320. PMC4584528 25837241

[55] De Lappe N, Garvey P, O’Connor J Ciprofloxacin-resistant Shigella sonnei associated with travel to India. Emerg Infect Dis. 2015;21:894–896. 25897625 10.3201/eid2105.141184 PMC4412218

[56] Centers for Disease Control Nationwide dissemination of multiply resistant *Shigella sonnei* following a commonsource outbreak. Morbid Mortal Wkly Rep. 1987;36:633–634. 3114612

[57] Centers for Disease Control *Shigella dysenteriae* type 1 – Guatemala. Morbid Mortal Wkly Rep. 1991;40:421–428. 2046650

[58] Liu Y, Walsh T, Ling-Xian B, et al Emergence of plasmid-mediated colistin resistance mechanism MCR-1 in animals and human beings in China: a microbiological and molecular biological study. Lancet Infect Dis. 2016;16:161–168. 26603172 10.1016/S1473-3099(15)00424-7

[59] Li B, Ni Y, Sun J, et al Molecular characterization of the extended-spectrum beta-lactamase (ESBL)-producing Shigella spp. in Shanghai. Eur J Clin Microbiol. Infect. Dis. 2015;34:447–451. 10.1007/s10096-014-2244-2 25252628

[60] Sabra G, Kattar M, Abi-Rached R, et al Molecular characterization of ESBL-producing Shigella sonnei isolates from patients with bacilliary dysentery in Lebanon. J Infect Dis. Dev Ctries. 2009;3:300–305. 10.3855/jidc.128 19759494

